# Bacteria can mobilize nematode-trapping fungi to kill nematodes

**DOI:** 10.1038/ncomms6776

**Published:** 2014-12-16

**Authors:** Xin Wang, Guo-Hong Li, Cheng-Gang Zou, Xing-Lai Ji, Tong Liu, Pei-Ji Zhao, Lian-Ming Liang, Jian-Ping Xu, Zhi-Qiang An, Xi Zheng, Yue-Ke Qin, Meng-Qing Tian, You-Yao Xu, Yi-Cheng Ma, Ze-Fen Yu, Xiao-Wei Huang, Shu-Qun Liu, Xue-Mei Niu, Jin-Kui Yang, Ying Huang, Ke-Qin Zhang

**Affiliations:** 1 Laboratory for Conservation and Utilization of Bio-Resources, Yunnan University, Kunming, Yunnan 650091, China; 2 State Key Laboratory of Phytochemistry and Plant Resources in West China, Kunming Institute of Botany, Chinese Academy of Sciences, Kunming, Yunnan 650201, China; 3 Department of Biology, McMaster University, Hamilton, Ontario, Canada L8S 4K1; 4 Texas Therapeutics Institute, The Brown Foundation Institute of Molecular Medicine, University of Texas Health Science Center, Texas 77030, USA

## Abstract

In their natural habitat, bacteria are consumed by bacterivorous nematodes; however, they are not simply passive preys. Here we report a defensive mechanism used by certain bacteria to mobilize nematode-trapping fungi to kill nematodes. These bacteria release urea, which triggers a lifestyle switch in the fungus *Arthrobotrys oligospora* from saprophytic to nematode–predatory form; this predacious form is characterized by formation of specialized cellular structures or ‘traps’. The bacteria significantly promote the elimination of nematodes by *A. oligospora*. Disruption of genes involved in urea transport and metabolism in *A. oligospora* abolishes the urea-induced trap formation. Furthermore, the urea metabolite ammonia functions as a signal molecule in the fungus to initiate the lifestyle switch to form trap structures. Our findings highlight the importance of multiple predator–prey interactions in prey defense mechanisms.

## Abstract (web summary)

Certain soil fungi form specialized cellular structures or 'traps' to feed on nematodes, which in turn eat bacteria. Here, the authors show that urea released from bacteria induces trap formation in the fungi and this promotes nematode elimination.

## Graphical abstract

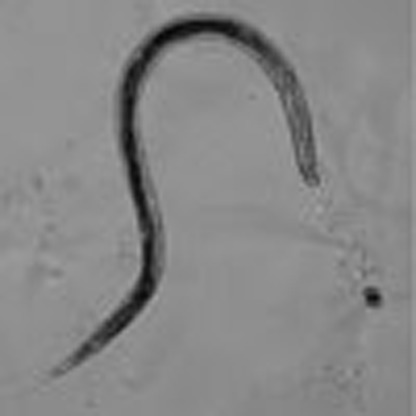

In natural ecosystems, bacterial communities play an important role in nutrient cycling, primary production and decomposition^1^. Bacteria are also heavily consumed by bacterivorous nematodes that are present in compost, soil and aquatic systems^1^,^2^. Thus, aside from competing for nutrients among themselves, bacteria experience significant predation pressure from nematodes. To counter predatory attacks from the metazoan enemies, bacteria have evolved a variety of defense mechanisms^3^. First, many bacteria can produce and secrete toxins. For instance, the bacterium *Pseudomonas fluorescens* secretes secondary metabolites to repel nematodes^4^, whereas *P. aeruginosa* produces phenazine compounds and cyanide to kill nematodes^5^,^6^. Second, the secretion of extracellular proteases contributes to the nematode toxicity of the bacteria *Vibrio cholerae* and *Bacillus nematocida* against nematodes^7^,^8^. Third, morphological adaptations by bacteria are also a common defense mechanism. For example, biofilm formation by the bacterium *Yersinia pestis* can prevent or reduce ingestion by nematodes (and other predators)^9^,^10^. These specific strategies provide certain bacteria an advantage against their nematode predators.

These bacterial defense mechanisms in turn create selective pressure on nematodes, resulting in predator–prey arms race. For example, nematodes have evolved to avoid the toxic/pathogenic bacteria and forage for nontoxic bacteria to support their growth and reproduction^11^. Currently, the most detailed data on nematode behavioral responses to bacteria have come from the model nematode *Caenorhabditis elegans*^12^. Although *C. elegans* has a relatively simple nervous system, its sensory neurons can detect many environmental cues. When encountering bacterial pathogens, *C. elegans* exhibits an avoidance behaviour and escapes^13^,^14^,^15^. In the presence of nontoxic bacteria, *C. elegans* can recognize and hunt for them as food sources. However, it remains unclear whether counter response strategies exist in these nontoxic bacteria.

In natural environments, the nematode-trapping fungi can trap and kill nematodes. These fungi have two lifestyle phases: saprophytic and predacious^16^,^17^. In the absence of nematodes, these fungi live as saprophytes. Upon encountering nematodes, these fungi enter the predacious stage by producing specialized predatory devices to capture and kill nematodes. Earlier studies have demonstrated that nematodes produce signal substances to promote trap formation in these fungi^18^, but the chemical compositions of these substances remain largely unknown. A recent study has reported that ascarosides, a conserved family of signalling molecules that are widely biosynthesized among nematodes^19^, induce trap formation in nematode-trapping fungi^20^. Aside from ascarosides, diffusible compounds from cow dung have also shown to induce lifestyle transitions in the model nematode-trapping fungus *Arthrobotrys oligospora*, which produces three-dimensional (3D) adhesive networks^21^.

We hypothesized that the diffusible components from cow dung could have a bacterial origin. Here we report that urea released from bacteria triggers a lifestyle switch in the fungus *A. oligospora* from saprophytic to predacious stage. Ammonia, a product of urea degradation, in turn serves as a signal molecule to initiate this lifestyle switch. These findings provide a striking example of multiple predator–prey interactions, which play an important role for modulating the composition and population dynamics in nature.

## Results

### Bacteria in cow dung are capable of inducing trap formation

**Figure 1:**
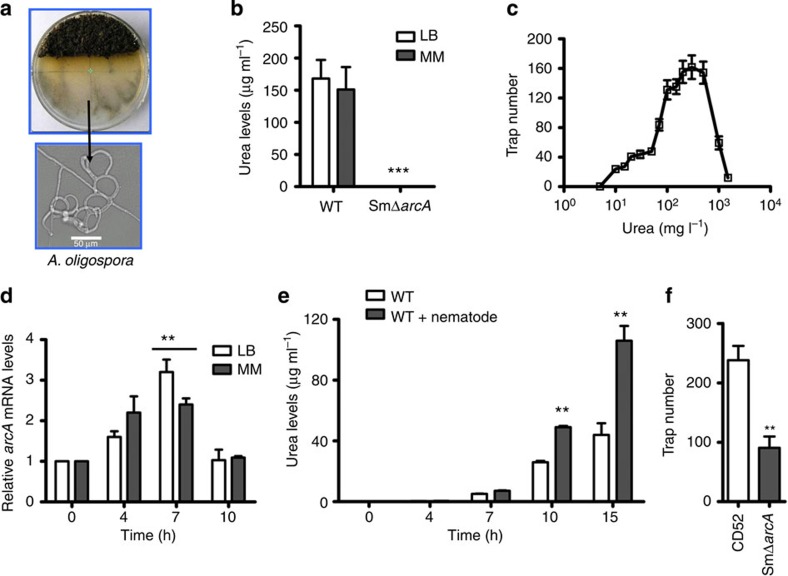
Urea produced by bacteria in cow dung functions as a signal molecule to induce trap formation in *A. oligospora.* (**a**) Trap formation in *A. oligospora* in the vicinity of cow dung. Cow dung (brown area in the bottom of the plate) was placed on a water agar plate. After conidia of *A. oligospora* were added at position 2 cm away from dung, traps were produced (arrow). (**b**) Deletion of *arcA* completely abolished the production of urea in *S. maltophila* CD52 in LB medium and MM, respectively. Data are expressed as mean±s.d. of three independent experiments. ***P<0.001 versus CD52 (*t-*test). (**c**) Effect of urea concentration on trap formation in *A. oligospora* at 72 h. (**d**) Expression of *arcA* in *S. maltophila* was induced by the addition of nematodes in LB medium and MM. Data are expressed as mean±s.d. of three independent experiments. **P<0.01 versus control (0 h; *t-*test). (**e**) The urea levels in the supernatant in *S. maltophila* were elevated by nematodes. Data are expressed as mean±s.d. of three independent experiments. **P<0.01 versus wild-type (WT) only (*t-*test). (**f**) Deletion of *arcA* significantly suppressed *S. maltophila*-mediated trap formation in *A. oligospora*. Data are expressed as mean±s.d. of three independent experiments. **P<0.01 versus CD52 (*t-*test).

Consistent with a previous observation, we found that fresh cow dung induced trap formation in *A. oligospora* on water agar plates (Fig. 1a). However, autoclaved dung samples induced fewer traps than fresh samples (Supplementary Fig. 1). As bacteria constitute a significant portion of the cow dung biota, these results suggest that active metabolites from bacteria in cow dung may serve as inducers of trap formation in *A. oligospora*. To test this idea, we first identified the specific bacteria in cow dung that can induce trap formation in *A. oligospora*. The aqueous suspension of fresh dung was serially diluted by 10-folds in sterilized water and spread-plated on nutrient Luria-Bertani broth (LB) agar. After 1 day of incubation, a variety of bacterial colonies appeared and 126 random colonies (named CD1 to CD126) were subcultured and analysed. 16S rRNA gene analysis clustered the 126 isolates into 18 genera (Supplementary Table 1). Of these isolates, supernatants from 55 isolates in LB broth medium could induce trap formation in *A. oligospora*. Three *Stenotrophomonas maltophilia* isolates (CD8, CD52 and CD103) were the most efficient inducers. In contrast, the supernatants of the remaining 71 isolates did not induce trap formation. These results indicate that some bacterial isolates obtained from cow dung can induce trap formation in *A. oligospora*.

### Identification of urea as the trap inducer in *A. oligospora*

Of the 55 isolates capable of inducing trap formation in *A. oligospora*, three *S. maltophilia* isolates (CD8, CD52 and CD103) were the most efficient ones. To determine the compound(s) secreted from the bacteria that induced trap formation in *A. oligospora*, the extract from fermentation supernatant of *S. maltophilia* CD52 in LB medium was isolated by silica gel G column and Sephadex LH-20 column chromatography. A candidate compound was obtained by activity-guided isolation, and further identified as urea with mass spectrometry, nuclear magnetic resonance and elemental analysis (Supplementary Fig. 2a–c; Fig. 1b). Urea was also found in the fermentation supernatant of *S. maltophilia* CD52 in a low-nutrient minimal medium (MM; Fig. 1b). These results suggest that urea is produced by *S. maltophilia* CD52 in both nutrient-rich and -deficient medium. Furthermore, urea was detected in the fermentation supernatants from the other 54 isolates that showed trap-inducing activity, but not from the remaining 71 isolates (Supplementary Table 1).

The observation that urea secreted by bacteria could induce fungal trap formation was further confirmed by analysing commercial ultrapure grade urea on water agar plates. After 72-h inoculation, urea significantly induced trap formation in *A. oligospora* in a dose-dependent manner, reaching maximum induction of trap formation at 300 mg l^−1^ (Fig. 1c). Interestingly, at the high concentration of 1,500 mg l^−1^, urea completely abolished the trap formation (Fig. 1c). Recently, Hsueh *et al.*^20^ have demonstrated that ascarosides produced by *C. elegans* can induce trap formation in nematode-trapping fungi. However, trap induction by ascarosides was completely suppressed in a medium containing 0.5% (5,000 mg l^−1^) of (NH_4_)_2_SO_4_. Thus, the results from our study and that of Hsueh *et al.*^20^ are consistent with the hypothesis that trap formation is inhibited under nitrogen-rich conditions. Next, we determined whether urea existed in the exudation of cow dung and found that the concentration of urea in exudation of cow dung was 151 mg l^−1^. Furthermore, the exudation of cow dung significantly induced the trap formation in *A. oligospora* (Supplementary Fig. 3).

In bacteria, urea is generated mainly through arginine catabolism in which arginase converts arginine into ornithine and urea in the last step of the urea cycle^22^. We randomly selected three urea-producing (CD52, CD82 and CD101) and three non-urea-producing bacteria (CD1, CD93 and CD102) that were identified at the species level, and tested whether putative *arcA* genes encoding arginases exist in the genomes of these bacteria and whether they are expressed. We amplified putative *arcA* genes from the genomic DNA and mRNA, respectively, by PCR using two degenerate primers. The amplified fragments were obtained from the genomic DNA of all the bacteria (Supplementary Fig. 4). DNA sequencing demonstrated that these fragments contain the *arcA* genes. In contrast, the amplified fragments were only obtained from the mRNA of the three urea-producing bacteria, but not of the non-urea-producing bacteria (Supplementary Fig. 4). The results suggest that the *arcA* genes might be induced under certain conditions in these non-urea-producing bacteria.

To determine whether arginase was required for urea production in *S. maltophila* CD52, we deleted the *arcA* gene in this strain (Supplementary Fig. 5a,b). As expected, no urea was detected in the fermentation supernatant from the SMΔ*arcA* mutant (Fig. 1b). We found that the expression of *arcA* in *S. maltophila* CD52 was significantly upregulated at 7 h after exposure to the nematode *C. elegans* in both LB medium and MM (Fig. 1d). Consistently, the urea levels in the supernatant were also markedly increased at 10 h after exposure to *C. elegans* in the LB medium (Fig. 1e). Finally, the deletion of *arcA* significantly suppressed the ability of *S. maltophila* CD52 to induce trap formation in *A. oligospora* (Fig. 1f). These results suggest that urea produced from bacteria induces trap formation in *A. oligospora*.

**Figure 2:**
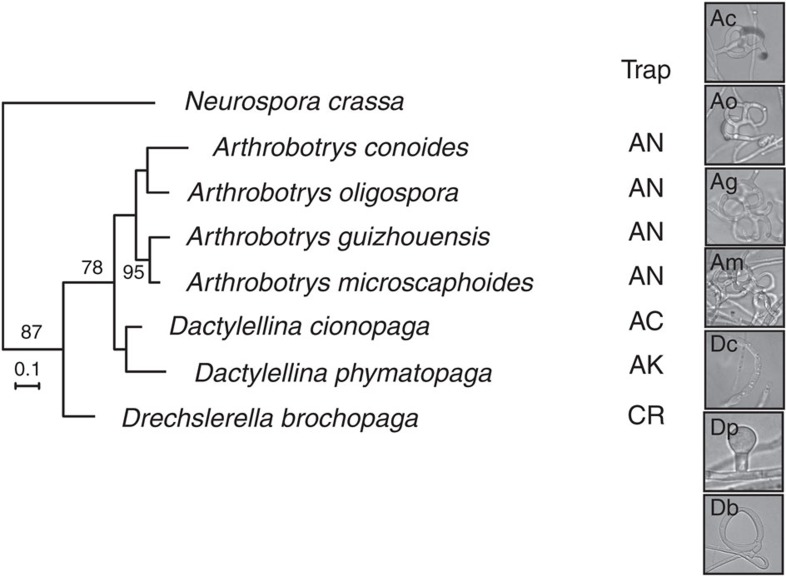
Urea-induced morphogenesis is common in nematode-trapping fungi. Bayesian inference phylogeny of the ITS regions of nematode-trapping fungi in the family Orbiliaceae, Ascomycota. The model filamentous fungus *Neurospora crassa* is used as outgroup. Trapping structures are AC, adhesive columns; AK, adhesive knobs; AN, adhesive networks and CR, constricting rings.

To investigate whether urea could induce trap formation in other nematode-trapping fungi, we tested urea responsiveness in additional species of the fungal family Orbiliaceae, Ascomycota. These fungi can produce a variety of trap structures including 3D adhesive networks, adhesive knobs, adhesive columns and constricting rings (Supplementary Table 2). We found that urea induced trap structures in 7 of the 31 species under the tested conditions (Fig. 2). For the remaining 24 species, it is likely that they respond to other signal molecules yet to be identified. The fact that the trap structures produced by these species cover most of the trap types suggests that urea responsiveness is common, but not conserved, among nematode-trapping fungi.

### Urea uptake is crucial for trap formation in *A. oligospora*

**Figure 3:**
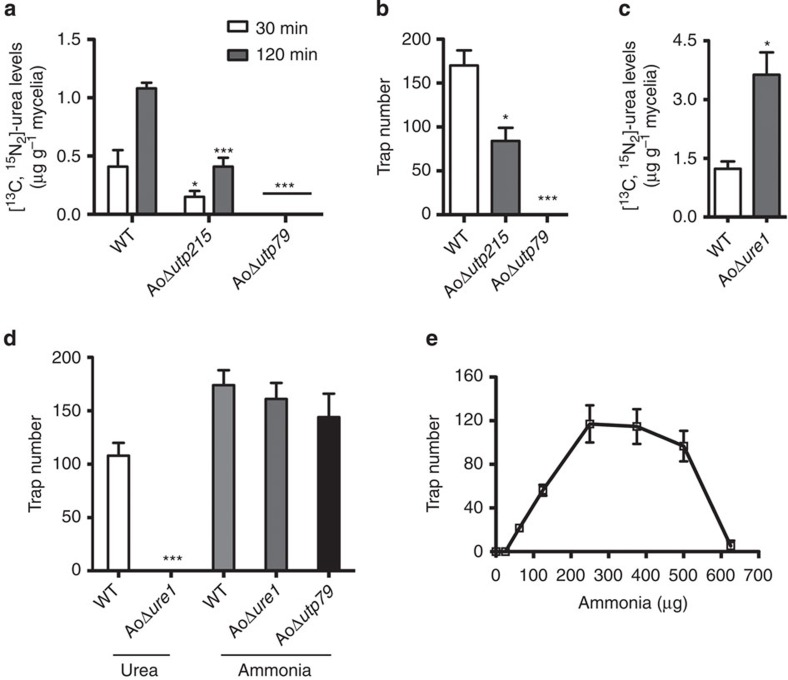
The transport and decomposition of urea are required for trap formation in *A. oligospora*. (**a**) [^13^C,^15^N_2_]-Urea uptake was completely abolished in the AoΔ*utp79* mutant. Disruption of *utp215* only partially impaired [^13^C,^15^N_2_]-urea uptake in *A. oligospora*. (**b**) Deletion of *utp79* eliminated the urea-induced trap formation in *A. oligospora*. In contrast, deletion of *utp215* partially inhibited urea-mediated trap formation. (**c**) Deletion of *ure1* led to urea accumulation in *A. oligospora*. [^13^C,^15^N_2_]-urea uptake was measured in the WT and AoΔ*ure1* mutant strains. (**d**) Deletion of *ure1* completely blocked the urea-induced trap formation in *A. oligospora.* However, ammonia rescued trap formation in the Ao*utp79* and AoΔ*ure1* mutants. (**e**) Application of ammonia induced trap formation (measured at 72 h). Data are expressed as mean±s.d. of three independent experiments. *P<0.05, and ***P<0.001 versus WT (*t-*test).

A number of transporters from fungi with high specificity for urea have been reported^23^,^24^,^25^. From the genome of *A. oligospora* ATCC24927 (ref. 17), we identified two putative urea transporter genes *utp79* and *utp215* (AOL_S00079g183r and AOL_s00215g323r). Phylogenetic analyses showed that these two urea transporters from *A. oligospora* were clustered into two separate groups (Supplementary Fig. 6). To determine whether Utp79 and Utp215 are the active urea transporters in *A. oligospora*, we deleted the two genes by homologous recombination (Supplementary Fig. 7a–c). Colony growth of the AoΔ*utp215* mutant strain was similar to that of the wild-type strain on potato dextrose agar (PDA) or corn meal agar (CMA), whereas the AoΔ*utp79* mutant strain grew slower than the wild-type strain on PDA, but not on CMA (Supplementary Fig. 8). Next, we measured urea uptake using [^13^C, ^15^N_2_]-urea as a tracer. The [^13^C, ^15^N_2_]-urea uptake was completely abolished when *utp79* was disrupted, whereas the disruption of *utp215* showed only partially impaired [^13^C, ^15^N_2_]-urea uptake (Fig. 3a). Importantly, the disruption of *utp79* completely blocked the urea-induced trap formation, whereas the trap formation was only partially suppressed in the AoΔ*utp215* mutant (Fig. 3b). These results indicate that urea needs to be transported into fungal cells to mediate trap formation in *A. oligospora*, mainly through Utp79.

### The urea metabolite ammonia functions downstream to induce trap formation

Generally, one urea molecule is converted to two ammonia molecules and one carbon dioxide molecule. From the genome of *A. oligospora*^17^, a putative urease gene, namely *ure1* (AOL_s00080g26r), was retrieved and analysed (Supplementary Fig. 9). We deleted *ure1* and found that the AoΔ*ure1* mutant strain grew slower than the wild-type strain on PDA and CMA (Supplementary Figs 7d and 8). Deletion of *ure1* resulted in significant intracellular accumulation of [^13^C, ^15^N_2_]-urea in *A. oligospora* (Fig. 3c). However, increased extracellular urea concentration (up to 300 mg l^−1^) failed to induce trap formation in the AoΔ*ure1* mutant (Fig. 3d). These data suggest that urea breakdown product(s) is crucial for the induction of trap formation.

We next investigated which of the two urea-breakdown products serves as the signal molecule to promote trap formation in *A. oligospora*. Unlike in *Candida albicans* and *Cryptococus neoformans* where carbon dioxide was effective at inducing morphological change^26^,^27^, carbon dioxide at a range of concentrations (1, 2.5 and 5%) failed to induce trap formation in *A. oligospora*. In contrast, ammonia effectively elicited trap formation in the wild-type strain (Fig. 3d). Similar to the response to urea, trap formation in *A. oligospora* responded to ammonia in a dose-dependent manner, reaching maximum induction of trap formation at 250 μg, whereas at 625-μg concentration, ammonia suppressed the trap formation (Fig. 3e). Furthermore, the addition of ammonia to both AoΔ*utp79* and AoΔ*ure1* mutant strains restored their trap formation (Fig. 3d), indicating that the urea breakdown product ammonia is the signal to induce trap formation.

### Urea promotes the pathogenicity of *A. oligospora*

**Figure 4:**
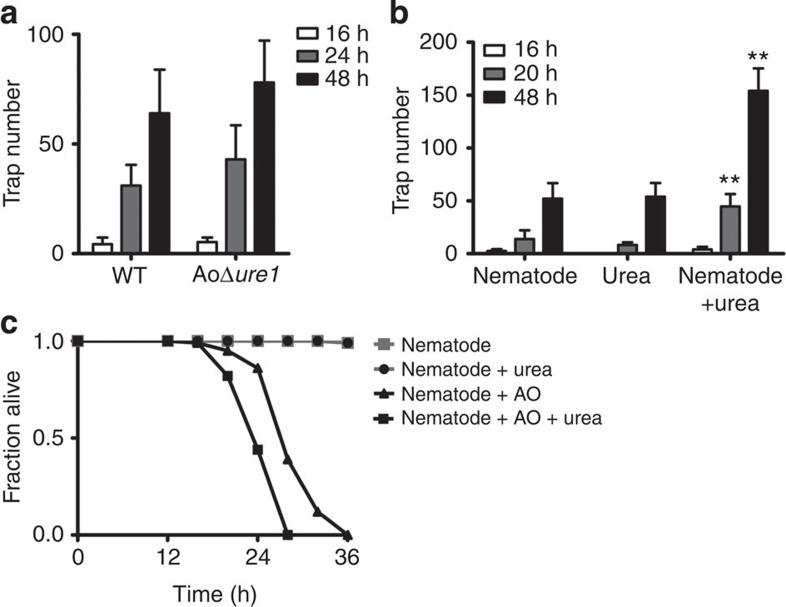
Urea reduces the survival of nematodes in the presence of *A. oligospora.* (**a**) The number of traps in WT and AoΔ*ure1* mutant strains in the presence of nematodes. Data are expressed as mean±s.d. of three independent experiments. (**b**) Urea promoted nematode-induced trap formation in WT strain. Data are expressed as mean±s.d. of three independent experiments. **P<0.01 versus control (without urea; *t-*test). (**c**) Urea reduced the survival of nematodes after *A. oligospora* (AO) infection. **P<0.01 versus AO without urea (*t-*test).

It has been reported that ascarosides secreted by nematodes can induce trap formation in nematode-trapping fungi^20^. We asked whether the bacteria/urea- and the nematode/ascaroside-induced traps share the same pathway in *A. oligospora*. To answer this question, we determined whether nematode induces trap formation in the AoΔ*ure1* mutant. We found that the number of traps in the AoΔ*ure1* mutant was comparable to that in wild-type strain in the presence of nematodes (Fig. 4a).

Next, we determined the effect of urea on nematode-induced trap formation. Nematodes and urea (100 mg l^−1^) induced trap formation in *A. oligospora* at 16, 20 and 48 h, respectively (Fig. 4b). Although the addition of urea did not shorten the initiation time of trap formation, it significantly increased the number of traps induced by nematodes (Fig. 4b). Urea and nematodes together led to a threefold increase in the number of traps, compared with that of urea or nematodes alone. Thus, nematode and urea exhibited synergistic effect on trap formation. Meanwhile, we found that urea markedly accelerated the elimination of nematodes mediated by *A. oligospora* (Fig. 4c). These results suggest that the bacteria/urea signalling induces trap formation via a mechanism different from the nematode/ascaroside signalling pathway.

### Tripartite relationships in soil

**Figure 5:**
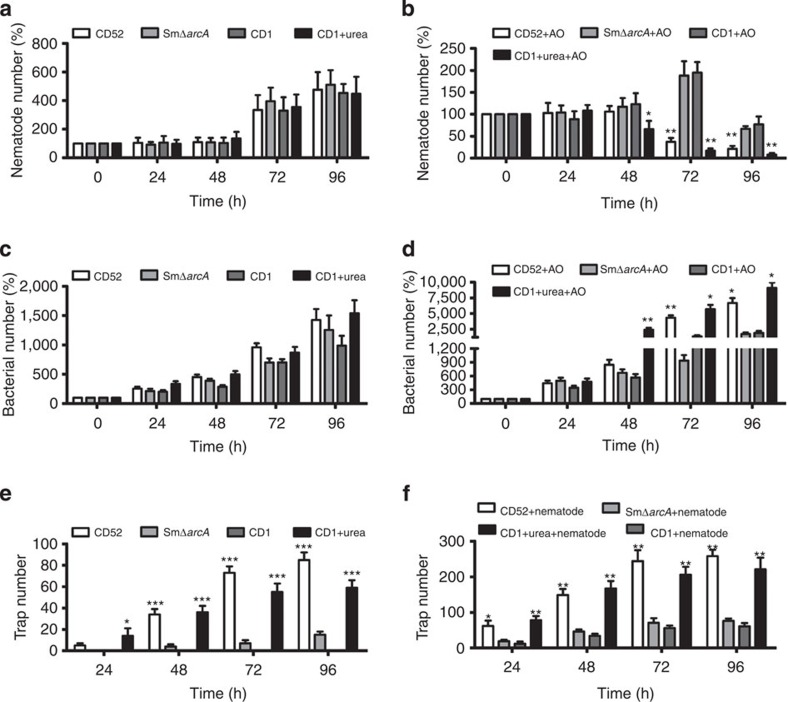
The relationship among bacteria, nematodes and fungi in the soil. (**a**) In soil without *A. oligospora*, the number of worms steadily increased in the presence of *S. maltophila* CD52, SmΔ*arcA* mutant, non-urea-producing bacterium *B. amyloliquefaciens* CD1 and CD1 plus urea (5 mg). (**b**) In soil with *A. oligospora*, the survival rates of worms were significantly lower in the presence of *S. maltophila* CD52 and CD1 plus urea than those in the presence of SmΔ*arcA* mutant or *B. amyloliquefaciens* CD1. The data are expressed as percent change from control (0 h). (**c**) In soil without *A. oligospora*, all the four groups exhibited a comparable number of bacteria in the presence of worms. (**d**) In soil with *A. oligospora*, the number of CD52 and CD1 (CD1 plus urea group) was much higher than that of Sm*ΔarcA* mutant and CD1 (CD1 alone group) in the presence of worms. (**e**,**f**) The number of traps in plates containing CD52 and CD1 plus urea was significantly higher than that in plates containing Sm*ΔarcA* mutant and CD1 in the absence (**e**) or presence (**f**) of worms. *P<0.05, **P<0.01 and ***P<0.001, CD52 versus SmΔ*arcA* or CD1+urea versus CD1 (*t-*test).

In order to investigate the relationship among bacteria, nematodes and nematode-trapping fungi in the soil, we first determined the reproduction of worms in four groups containing *S. maltophilia* CD52, SmΔ*arcA* mutant, *B. amyloliquefaciens* CD1 and CD1 plus urea (5 mg), respectively. *B. amyloliquefaciens* CD1 is a non-urea-producing bacterium isolated from cow dung (Supplementary Table 1). As expected, the number of nematodes was all similarly increased at 72 and 96 h in the absence of *A. oligospora* (Fig. 5a). Next, we determined the effect of these bacteria on *A. oligospora*–nematode interaction. In the presence of *A. oligospora,* the soil pre-inoculated with CD1 plus urea or CD52, the number of worms started to decrease significantly at 72 h and almost all worms died at 96 h (Fig. 5b). In contrast, in soil containing SmΔ*arcA* mutant and *A. oligospora*, the number of nematodes increased by 1.8-fold at 72 h and began to decrease at 96 h. Similar results were obtained in soil pre-inoculated with CD1 and *A. oligospora* (Fig. 5b). Together, our results indicate that urea-producing bacteria promote the elimination of nematodes by *A. oligospora*.

Next, we determined the colony-forming units of bacteria in soil. We found that all four groups exhibited a comparable number of bacteria in the presence of worms alone (Fig. 5c). The number of CD52 or CD1 (CD1 plus urea group) was much higher than that of Sm*ΔarcA* mutant or CD1 (CD1 alone group) in the presence of worms and *A. oligospora* (Fig. 5d). As it is difficult to calculate the number of traps in soil, we performed similar experiments in agar plates. In the absence of nematodes, the number of traps in plates containing CD52 or CD1 plus urea increased gradually, and reached the highest level at 72 h (Fig. 5e). Although traps were induced by the Sm*ΔarcA* mutant, the number of traps was significantly lower than that induced by CD52 or CD1 plus urea. In contrast, CD1 did not induce trap formation. Meanwhile, in the presence of worms, the number of traps in plates containing either CD52 or CD1 plus urea was significantly higher than that in plates containing SmΔ*arcA* mutant or CD1 without urea (Fig. 5f). Taken together, our results indicate that urea-producing bacteria mobilize the nematode-trapping fungus *A. oligospora* and provide a protection against their nematode predators.

### Urea diffuses readily in soil

It has been shown that the volatiles released by plants can attract insect predators from long distances^28^,^29^,^30^,^31^. In contrast, the nonvolatile urea is likely to function as a short-range signal molecule. To examine whether urea diffuses in moist sand, 5 mg of urea was added into a spot in a sand-filled dish. Every 30 min, samples were taken at a distance of 8 cm from this spot by a small hole puncher and analysed by liquid chromatography–mass spectrometry (LC-MS). Urea was detected at the first 30 min after introduction in sand. The amount of urea attained the highest level at 120 min, and declined to the basal level at 180 min (Supplementary Fig. 10). The rapid diffusion of urea in moist sand seems to make it suitable as a ground signal.

## Discussion

**Figure 6:**
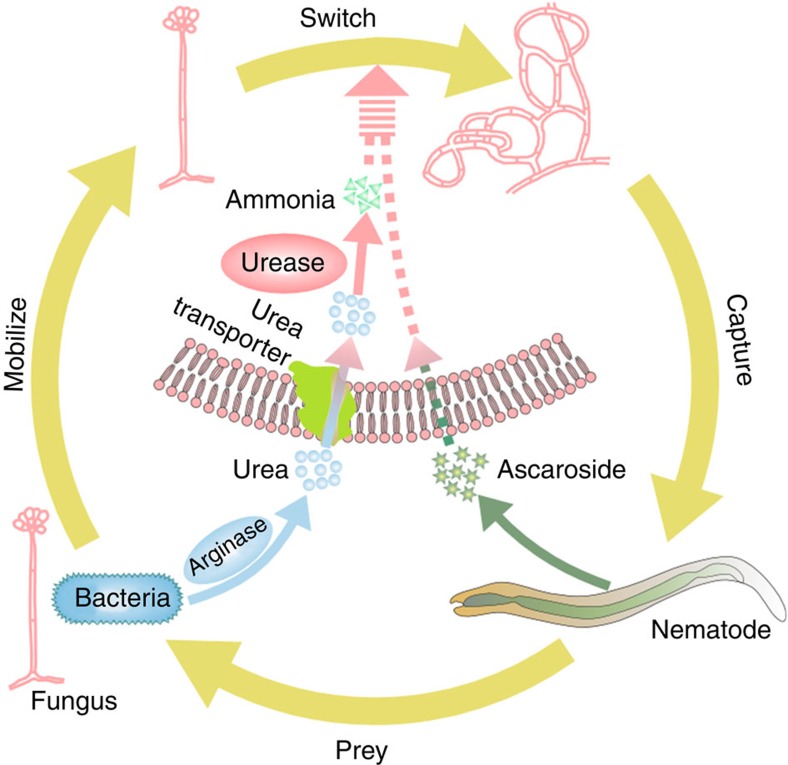
A model for trap formation by nematodes and bacteria. When grazed by nematodes, bacteria increase the production and release of urea by upregulating the expression of arginase. Secreted urea is then taken up by the mycelia of nematode-trapping fungi nearby via a urea transporter, and eventually catabolized to ammonia by urease within the fungi. Ammonia in turn initiates the lifestyle switch to form trap structures. Meanwhile, ascarosides released by nematodes are transported into the mycelia of nematode-trapping fungi by unknown proteins, and induce trap formation directly. The bacteria/urea- and the nematode/ascaroside-mediated signalling pathways exhibit significant synergistic effects on trap formation, resulting in the eventual capture and death of the nematodes.

As major components of the soil ecosystem, bacterivorous nematodes and bacteria interact with each other and have evolved a diversity of mechanisms to counter balance each other^1^,^2^,^3^. However, little is known about the mechanisms by which the nonpathogenic bacteria protect themselves against the nematode grazers. Our results demonstrate that certain bacteria can mobilize nematode-trapping fungi to reduce predation pressure by producing and releasing urea. The urea metabolite ammonia functions as a signal molecule to accelerate the switch in nematode-trapping fungi from saprophytic to predacious lifestyle, which is crucial for capturing nematodes (Fig. 6).

An indirect defense mechanism against predators has been reported in plants^28^,^29^,^30^,^31^ and insects^32^,^33^. When bitten by herbivorous insects, the insect-damaged leaves and roots of plants release a large amount of volatile chemicals. These chemicals attract both parasitic and predatory insects, which are natural enemies of the herbivores, thus enabling them to locate and attack the herbivores^28^. Similarly, the termite (*Coptotermes formosanus*) uses its faeces to build its nest structure that provide nutrition to support the growth of a *Streptomyces* bacterium^32^. The presence of *Streptomyces* in turn inhibits the growth of *Metarhizium anisopliae*, an entomopathogenic fungus, providing a significant survival benefit to the termites. Thus, such multispecies interactions are likely very common in natural ecosystems.

Unlike the volatiles released by plants to attract carnivores from long distances^28^,^29^,^30^,^31^, the bacteria release nonvolatile urea as an intermediate message to communicate with fungi nearby. The high solubility and rapid diffusion of urea make it a suitable molecular signal in the ground. As urea transport is critical for inducing trap formation in *A. oligospora*, urea appears to function intracellularly. After entering cells, urea is metabolized to carbon dioxide and ammonia by urease. Our finding reveals that ammonia, not carbon dioxide, from urea metabolism, functions as the downstream molecule to initiate the lifestyle switch in nematode-trapping fungi. Accumulating evidence has demonstrated that both ammonia and carbon dioxide can be used as signal molecules in morphogenetic transitions in the fungal kingdom. For instance, ammonia acts as a signal to promote the reprogramming of cellular metabolism, colony differentiation and the switch from pseudohyphal to yeast-like forms in yeast species *S. cerevisiae* and *C. mogii*^34^,^35^. Meanwhile, carbon dioxide derived from urea breakdown is known to mediate the switch from yeast to hypha form in *C. albicans* inside macrophages^26^, and to induce capsule production in *C. neoformans*^27^. Carbon dioxide triggers the yeast-to-hypha switch of *C. albicans* by activating the cAMP-dependent protein kinase A pathway^26^. At present, the molecular mechanisms by which ammonia controls these fungal morphogenetic transitions remain to be investigated.

On the basis of genomic and proteomic analyses, we have previously proposed a model for trap formation induced by nematodes in the nematode-trapping fungus *A. oligospora*. According to this model, multiple signal transduction pathways are activated by nematodes, involving a variety of cellular processes such as energy metabolism, biosynthesis of the cell wall, cell division, glycerol accumulation and peroxisome biogenesis. Recently, Hsueh *et al.*^20^ have identified that ascarosides produced by *C. elegans* are responsible for inducing trap formation in nematode-trapping fungi. However, our results demonstrate that nematodes can induce trap formation in the AoΔ*ure1* mutant, implicating that nematode-mediated trap formation is independent of ammonia. Thus, the bacteria/urea- and the nematode/ascaroside-mediated signalling pathways use different mechanisms to initiate the lifestyle switch in nematode-trapping fungi. Interestingly, the application of urea is sufficient to increase the number of traps induced by nematodes, suggesting that significant synergistic effects on trap formation exist between the two signalling pathways (Fig. 6). Furthermore, the bacteria/urea signalling markedly elicits the elimination of nematodes by *A. oligospora* in soil. These results suggest that these bacteria and nematode-trapping fungi form mutually beneficial relationships, thereby turning the nematodes from a bacteria hunter into a victim. The role reversals in multiple predator–prey interactions provide novel insights and add complexities to our understanding of the composition and population dynamics of microbial communities in natural ecosystems.

## Methods

### Induction of trap formation by cow dung

Fresh cow dung was collected from a cattle farm in Yunnan Agricultural University, Kunming, China. *A. oligospora* Fres. (ATCC24927) was obtained from American Type Culture Collection (ATCC). The induction of trap formation in *A. oligospora* by fresh or autoclaved dung was tested according to the method described previously^21^, with some modifications. Briefly, the cow dung (5 g) was diluted with 5 ml of water and placed on a water agar ~2.5 cm from the rim of a 90-mm-diameter plate. After pre-incubation for 7 days at 25 °C, 200 μl of conidial suspension with a concentration of ~5 × 10^4^ conidia per ml was spread over the plate and incubated at 25 °C. The traps were observed and scored using a light microscope.

### Induction of trap formation by bacteria

A total of 5 g fresh dung sample was added to 50 ml of sterile water and mixed thoroughly. The aqueous suspension of fresh dung was serially diluted by 10-folds in sterilized water and spread over LB agar plates. After 1 day of incubation at 30 °C, different colonies, named CD1 to CD126, were picked to obtain pure cultures. These colonies were cultured in 50 ml LB medium at 30 °C for 4 days and fermentation supernatants were collected. Two hundred microlitres of each supernatant mixed with fifty microlitres conidial suspension of *A. oligospora* with a concentration of ~5 × 10^4^ conidia per ml was spread over a water agar plate and incubated at 25 °C for 72 h. Trap numbers were counted in 10 low-power light microscope fields and reported as the average number per field of observation.

### Identification of bacteria isolated from cow dung

Bacterial genomic DNA was isolated using a commercial DNA isolation kit (Bioteke, Beijing, China). For identification of bacteria, the 16S rRNA gene sequences were amplified with the universal bacterial primers 8F (5′-AGAGTTTGATCCTGGCTCAG-3′) and 1492R (5′-GGTTACCTTGTTACGACTT-3′). PCR products were purified using the Gel Extraction & PCR Purification Combo Kit (Bioteke). The 16S rRNA genes were sequenced and analysed using the BLAST algorithm ( http://blast.ncbi.nlm.nih.gov/Blast.cgi).

### Isolation and identification of the active compound

After *S. maltophilia* CD52 was grown in 5 l of LB medium on a rotary shaker at 30 °C for 4 days, fermentation supernatant was collected and concentrated to final volume of 1 l. The supernatant was then extracted with equal volume of *n*-butanol. The *n*-butanol extract was concentrated to dryness in a rotary evaporator and the residue (10.4 g) was dissolved in methanol. Column chromatograph was performed on silica gel G column eluting with ethyl acetate/methanol (60:1–1:1) providing fractions A_1_–A_30_. Subsequently, these fractions were tested for their abilities to induce trap formation in *A. oligospora*. The active fraction A_3_ (1.62 g) was further separated and purified on a Sephadex LH-20 column (600 g) eluting with methanol to yield fractions A_3–1_–A_3–10_. We found that only the fraction A_3–2_ could induce trap formation. Then, the active fraction A_3–2_ (923 mg) was again purified on Sephadex LH-20 (200 g) eluting with methanol to obtain a single candidate active compound (426 mg). NMR experiment was carried out on Bruker Avance III-600 NMR spectrometers with tetramethylsilane as internal standard (Bruker Corporation, Fällanden, Switzerland). Electrospray ionisation mass spectrometry profiles were recorded on a Finnigan LCQ-Advantage mass spectrometer (Thermo Electron Corp., San Jose, CA, USA). Elemental analysis was measured on VARIO ELII element analyzer (Elementar Analysensysteme GmbH, Hanau, Germany).

### Detection of urea in supernatants

*S. maltophilia* CD 52 and SmΔ*arcA* mutant strains were grown in 100 ml LB medium at 30 °C for 4 days. The fermentations were extracted with equal volume of *n*-butanol. The *n*-butanol extracts were concentrated to dryness in rotary evaporator and the residues were dissolved in 10 ml methanol. Urea was determined using LC-MS with a Waters Series HPLC 2695 (Waters Corp., Milford, MA, USA) with Thermo Finnigan LCQ Advantage mass detector (ion trap; Thermo Electron Corp.). The instrument conditions were optimized as follows: spray voltage, 5.3 kV; capillary voltage, 3.00 V; capillary temperature, 250 °C. Samples were separated using a BDS HYPERSIL 5-μm pore size 4.6 mm × 250 mm column (Thermo Electron Corp.) with isocratic elution (5% methanol) at a flow rate of 200 μl min^−1^. In LC-MS analysis, urea (Sigma, St Louis, MO, USA) with m/z 61 [M+H]^+^ was used as an external standard. Retention time of urea was 15.00–16.10 min.

### Induction of trap formation by urea

For analysis of trap induction in nematode-trapping fungi by urea, 200 μl of conidial suspension with a concentration of ~5 × 10^4^ conidia per ml was spread over a water agar plate. After incubating with urea (0–1,500 mg l^−1^) for 72 h, traps were counted under a light microscope. For ammonia assays, 25% of ammonia solution (Shantou Xilong Chemical, Guangdong, China) were added into one compartment of a two-compartment Petri dish with 90 mm diameter, and 100 μl of conidial suspension with a concentration of ~5 × 10^4^ conidia per ml was spread over the other compartment containing water agar, respectively. Plates were immediately wrapped with Bemis ParafilmM sealing film (Bemis Flexible Packaging, Neenah, WI, USA) to prevent the escape of the volatiles. After incubating at 25 °C for 72 h, the traps were recorded under a light microscope. Three plates were performed per assay and all experiments were performed three times. Trap numbers were counted in 10 low-power light microscope fields, and reported as the average number per field of observation.

### Amplification of putative *arcA* genes

The genomic DNA of bacteria was extracted using the DNA isolation kit (Bioteke). Total RNA from bacteria was isolated using RNAiso Plus (Takara, Dalian, China). The putative *arcA* genes were amplified from the genomic DNA and mRNA using PCR. The primers used to amplify *arcA* genes were as follows: CD1F, 5′-ACCCGCACAGGACAACATA-3′, CD1R, 5′-GGAACCGCTCACTCTCAAT-3′; CD93F, 5′-CAGGAATAACATCACCACAGT-3′, CD93R, 5′-TGAAGCGGATATTGCCATAC-3′; CD102F, 5′-ATCCAAGCACTATGAAAAC-3′, CD102R, 5′-GGAGAATAACCACCAATGT-3′; CD52F, 5′-TCCCATTTCCGTATCCC-3′, CD52R, 5′-CCACGCCACCACTTCATC-3′; CD82F, 5′-ACTTCCAGCCGTATGACAG-3′, CD82R, 5′-ATTCACAACCGTTCGCATAG-3′; CD101F, 5′-ATCTCGGCGTCATCTGGTA-3′, CD101R, 5′-CGTCGTTCGGATCAAGTCC-3′. The primers for 16S rRNA gene were as follows: 533F, 5′-GTGCCAGCAGCCGCGGTAA-3′, 907R, 5′-CCGTCAATTCMTTTRAGTTT-3′.

### Deletion of *arcA* in *S. maltophilia*

The *S. maltophilia* CD52 *arcA* knockout vector was constructed by using the gene replacement vector pSUP202. Briefly, a 925-bp fragment spanning the entire coding region of the *arcA* gene, was amplified from genomic DNA of *S. maltophilia* CD52 strain by PCR using primers arcAF (5′-GAATTCATGGCCCATCCCATCTCCGTATC-3′) containing a *Bam*HI endonuclease recognition site and arcAR (5′-AAGCTTGCATCAGCGTGGACTTGCCGAAC-3′) containing a *Hind*III recognition site. The fragment was inserted into pMD-18T vector by T/A cloning, resulting in pMD-arcA. A 1,614-bp fragment of tetracycline (Tc) amplified from plasmid pRK415 with primers 415TcF (5′-CTGCAGAGTTTGCGTGTCGTCAGAC-3′) containing a *Pst*I recognition site and 415TcR (5′-GCATGCTCCTTACTGGGCTTTCTCA-3′) containing a *Sph*I recognition site. The fragment was inserted into pMD-18T vector by T/A cloning, resulting in pMD-Tc. The plasmids of pMD-arcA and pMD-Tc were digested with *Pst*I/*Sph*I, respectively. The Tc *Pst*I/*Sph*I fragment was cloned into the *Pst*I/*Sph*I gaped site of *arcA*, resulting in the pMD-arcA-Tc vector. Considering the size of the arcA-Tc fragment is close to that of the pMD-18T vector, the plasmid pMD-arcA-Tc was digested with *Dra*I. The fragment was then digested with *Bam*HI/*Hind*III and cloned into pSUP202. The final plasmid pSUP-arcA-Tc was introduced into *Escherichia coli* S17-1 (λpir) using an electroporation programme with a pulse charge at 2.5 kV and pulse time of 6 ms. The plasmid was then mobilized into *S. maltophilia* CD52 via conjugation.

Transconjugants were performed on LB agar supplemented with 50 μg ml^−1^ of tetracycline, 100 μg ml^−1^ of ampicillin and 50 μg ml^−1^ of kanamycin. The resistant colonies were selected into LB broth supplemented with 50 μg ml^−1^ of tetracycline and then incubated overnight at 30 °C. DNA from tetracycline-resistant colonies were isolated and confirmed using PCR. The primers used for PCR were arcAF and arcAR.

### Quantitative real-time PCR analysis

Total RNA from *S. maltophilia* CD52 was isolated using RNAiso Plus (Takara). Reverse transcription was conducted based on RNA extracted using PrimeScript RT reagent Kit with gDNA Eraser (Perfect Real Time; Takara) according to the manufacturer’s instructions. A real time-PCR analysis was performed with the Roche LightCycler 480 System (Roche Applied Science, Penzberg, Germany) using SYBR Premix-Ex Tag GC (Takara). The primers used for PCR were as follows: *arcA*: 5′-GTCGCTGGACATCGTTGAG-3′ (F), 5′-CGTGGACTTGCCGAACAG-3′ (R); *rpoD*: 5′-GGGCGAAGAAGGAAATGGTC-3′ (F), 5′-CAGGTGGCGTAGGTGGAGAA-3′ (R).

### Deletion of genes in *A. oligospora*

To delete *utp79*, two fragments (1,500 and 1,583 bp) were amplified with PCR using utp79-5F/utp79-5R and utp79-3F/utp79-3R, respectively. For deletion of *utp215*, two fragments (both of 1,674 bp) were amplified with PCR using utp215-5F/utp215-5R and utp215-3F/utp215-3R, respectively. For deletion of *ure1*, two fragments (both of 2,387 bp) were amplified with PCR using ure1-5F/ure1-5R and ure1-3F/ure1-3R. The sequences of these primers were as follows: utp79-5F, 5′-GTAACGCCAGGGTTTTCCCAGTCACGACGCAAAGCCGTTTATCAAGAAC-3′, utp79-5R, 5′-ATCCACTTAACGTTACTGAAATCTCCAACAGAATACCAGAACCGAGACC-3′; utp79-3F, 5′-CTCCTTCAATATCATCTTCTGTCTCCGACGTCAACTCCGTTATCAATCT-3′, utp79-3R, 5′-CGGATAACAATTTCACACAGGAAACAGCCATCCATCTAACCCCTCTTT-3′; utp215-5F, 5′-GTAACGCCAGGGTTTTCCCAGTCACGACGCGGTTGGCAAAGATAAGCAG-3′, utp215-5R, 5′-ATCCACTTAACGTTACTGAAATCTCCAACAGGCGGAGTGAGTGTAGTCG-3′; utp215-3F, 5′-CTCCTTCAATATCATCTTCTGTCTCCGACGATGTTTCGGCTGGTCTCGT-3′, utp215-3R, 5′-GCGGATAACAATTTCACACAGGAAACAGCTGTTGGATTTTGGGATAGGT-3′; ure1-5F, 5′-GTAACGCCAGGGTTTTCCCAGTCACGACGCATTATCGTCTCAACTACCC-3′, ure1-5R, 5′-ATCCACTTAACGTTACTGAAATCTCCAACTATTCATTTTATTTGTCCGC-3′; ure1-3F, 5′-CTCCTTCAATATCATCTTCTGTCTCCGACTTGTGGGTTGGTTTCTATG-3′, ure1-3R, 5′-GCGGATAACAATTTCACACAGGAAACAGCTGCTCTTTTCTTGCTTTCT-3′. The deletion cassettes were constructed by double crossing-over homologous recombination according to the method described previously^36^,^37^. The primers used for PCR were as follows: utp79F, 5′-CAGGAACCAATCCAAAGAGC-3′, utp79R, 5′-CAACCCAATAAAGTCGCACG-3′; utp215F, 5′-GCTGTGGTTCACTACCTTAC-3′, utp215R, 5′-ACACCCATCATCAAGTAGAG-3′; ure1F, 5′-AGTCGTAGCATTCGTCCCAG-3′, ure1R, 5′-ACCGTTCATCACCCCATTTC-3′.

### Uptake of [^13^C, ^15^N_2_]-urea

About 200 μl of conidial suspension of *A. oligospora* with a concentration of ~1 × 10^6^ conidia per ml was grown in potato dextrose broth medium. After 6 days, the mycelia were washed and re-suspended in water. [^13^C, ^15^N_2_]-Urea uptake was determined using MM containing 0.2 mg ml^−1^ [^13^C, ^15^N_2_]-urea (Sigma) at 25 °C, pH 6.8. After incubation for 30 and 120 min, mycelium was collected. The resulting mycelial pellet (100 mg) was washed with PBS five times. The pellet was ground with liquid nitrogen and extracted using 2 ml methanol. The extracts were then condensed to 100 μl. [^13^C, ^15^N_2_]-Urea was determined with LC-MS. For LC-MS quantification, [^13^C, ^15^N_2_]-urea with *m/z* 64 [M+H]^+^ was used as an external standard. The retention time of [^13^C, ^15^N_2_]-urea was 15.00–16.10 min. A calibration curve was constructed using known concentrations of urea ranging from 200 ng ml^−1^ to 10 mg ml^−1^. Each solution of 10 μl (2–100 ng) was injected on the column. All assays were performed in duplicates. Experimental data were calibrated and quantified using the Xcalibur Software (Thermo Finnigan). The calibration curves were constructed based on peak areas of the calibrated standards. Concentrations of all analytes were calculated from their peak areas against the calibration curves. The limit of detection for each analyte was determined as three times the signal-to-noise ratio. Recovery percent for each analyte in each matrix was obtained as the calculated concentration divided by the actual concentration.

### Induction of trap formation by nematodes

For analysis of trap formation induced by the nematode *C. elegans*, 200 μl of conidial suspension with a concentration of ~5 × 10^4^ conidia per ml was inoculated on water agar. After 2 days of growth, 80–100 adult nematodes were added to *A. oligospora* mycelia in the presence or absence of urea (100 mg l^−1^). After 16 h, mycelia were observed at time intervals under light microscope (Olympus, Tokyo, Japan).

### Interaction among bacteria, nematodes and fungi

Plates (35 mm) were first filled with sterilized soil (3 g). Bacterial suspension (500 μl; OD600=0.1), ~300 synchronized L3 *C. elegans* larvae or 100 μl of urea (50 mg ml^−1^), was added to each plate in the presence or absence of 200 μl conidia of *A. oligospora* (5 × 10^4^ conidia per ml). After inoculation for 24 h at 25 °C, the soil samples were collected at 24-h intervals. The soil samples were suspended in 10-ml sterile water. To count worm numbers, 1 ml of suspension of each treatment was taken and the number of worms was counted under a light microscope. To count colony-forming unit of bacteria, the suspension of each treatment was serially diluted by 10-folds in sterilized water and spread over LB agar plates. After 1 day of incubation at 30 °C, bacterial colonies were counted.

For measurement of traps in the presence of nematodes and bacteria, 200 μl conidia of *A. oligospora* (5 × 10^4^ conidia per ml) were pre-inoculated in water agar plates for 36 h. Then, 200 μl bacterial suspension (OD600=0.1) or 100 μl of urea (50 mg ml^−1^) with and without 60 synchronized L3 *C. elegans* larvae were added to water agar plates. After inoculation at 25 °C for 24 h, the traps were observed and scored using a light microscope at the indicated time points.

### Urea diffusion measurements

The diffusion of urea was determined by the method described by Rasmann *et al.*^30^, with some modifications. Briefly, a glass dish (30 cm in diameter, 8 cm deep) was filled with a 3-cm layer of moist sand (10% water). With a micropipette, 5 mg of urea in 100 μl of water was placed 1 cm deep in the sand at the centre of the dish. At a distance of 8 cm from this spot, the samples were taken by a small hole puncher (0.8 cm in diameter and 1 cm deep) every 30 min. The urea in the sand was analysed with LC-MS.

### Statistics

Differences in gene expression, trap formation, urea content and nematode number were assessed by the Student’s *t-*test. Data were analysed using the SPSS11.0 software (SPSS Inc.).

## Author contributions

K.-Q.Z., C.-G.Z. and G.-H.L. designed the experiments and analysed the data. X.W., G.-H.L., T.L., P.-J.Z., L.-M.L., X.Z., Y.-K.Q., M.-Q.T. and Y.-Y.X. performed the experiments. X.-L.J., J.-P.X., Z.-Q.A., Y.-C.M., Z.-F.Y., X.-W.H., S.-Q.L., X.-M.N. and J.-K.Y. interpreted the data, Y.H. provided the key reagents. K.-Q.Z., C.-G.Z., X.W., J.-P.X. and G.-H.L. wrote the manuscript. All authors discussed the results and commented on the manuscript.

## Additional information

**How to cite this article**: Wang, X. *et al.* Bacteria can mobilize nematode-trapping fungi to kill nematodes. *Nat. Commun.* 5:5776 doi: 10.1038/ncomms6776 (2014).

## Supplementary Material

Supplementary Information Supplementary Figures 1-10 and Supplementary Tables 1-2
