# Supplementary material for: Bacteria can mobilize nematode-trapping fungi to kill nematodes
Source: Nat Commun. 2014 Dec 16;5:5776. doi: 10.1038/ncomms6776 (PMC4275587; doi:10.1038/ncomms6776)
Supplement: Supplementary Information — Supplementary Figures 1-10 and Supplementary Tables 1-2 [file ncomms6776-s1.pdf]

Supplementary Figures

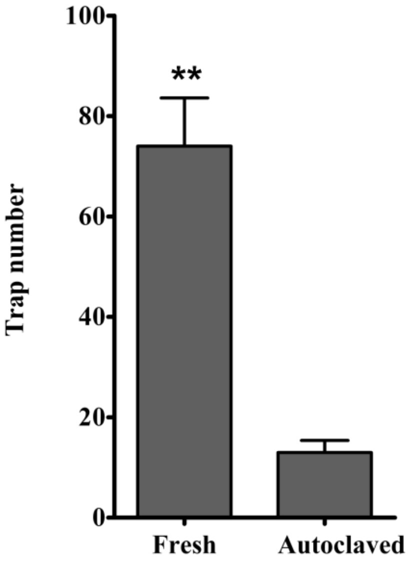

Supplementary Figure 1. Comparison of trap formation by fresh and autoclaved dung samples. The fresh or autoclaved dung was diluted with water and placed on a water agar plate within the scope of 2.5 cm from the rim of the plate with 90 mm diameter. After pre-incubation for seven days at 25 °C, conidial suspension of *A. oligospora* was spread over the plate and incubated at 25 °C. The traps were observed and scored using a light microscope. ** $P < 0.01$ versus autoclaved group (*t*-test).

a

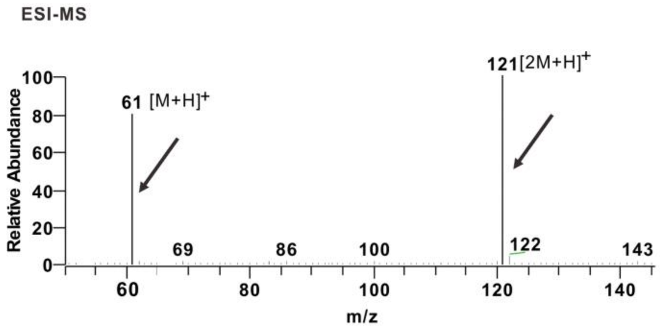

b

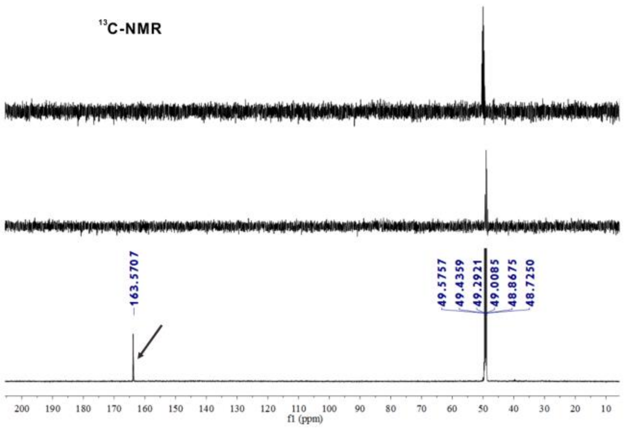

c

Elemental analysis

Elemental Composition	Content (%)
N	45.84
C	21.36
H	6.201

Supplementary Figure 2. Identification of urea from fermentation supernatant of *S. maltophilia* CD52. Fermentation supernatant from *S. maltophilia* CD52 was collected, and extracted with *n*-butanol. The *n*-butanol extract was concentrated to dryness, and dissolved in methanol. Then the extract was isolated by silica gel G column and Sephadex LH-20 column chromatography. A candidate compound was obtained by activity-guided isolation, and further identified as urea by mass spectra (**a**), ^{13}C-NMR (**b**), and elemental analysis (**c**).

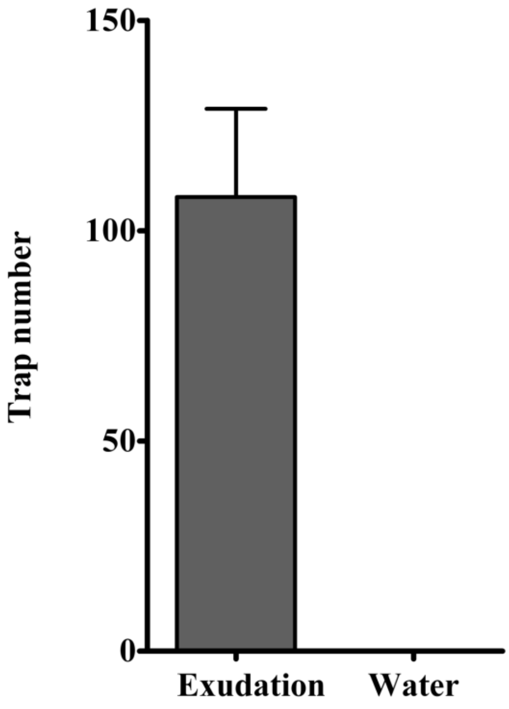

Supplementary Figure 3. The exudation induces trap formation. The fresh cow dung was diluted with equal volume water and placed on a water agar plate. Then conidial suspension was spread over the plate and incubated at 25 °C. The traps were observed and scored using a light microscope.

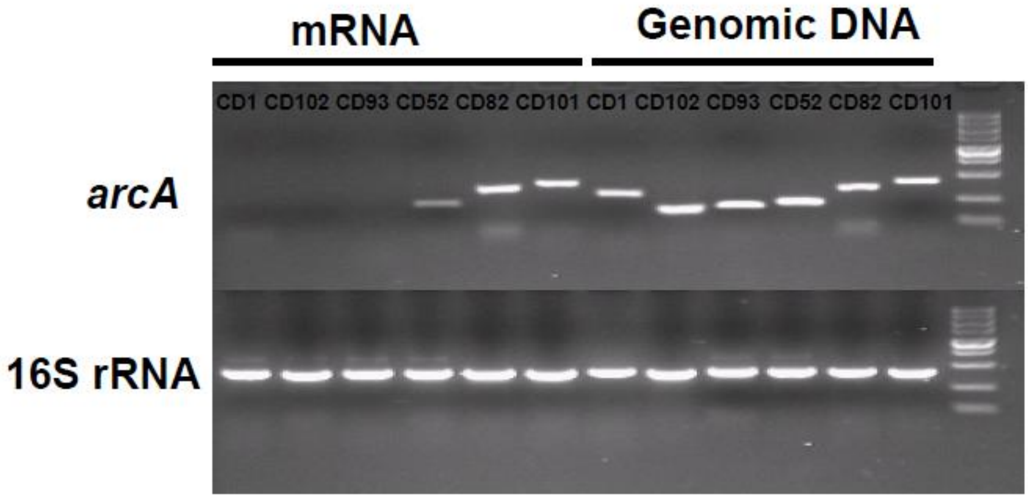

Supplementary Figure 4. The amplified fragments of putative *arcA* genes are obtained from the genomic DNA and mRNA by RT-PCR. CD52, CD82, and CD101, urea-producing bacteria; CD1, CD93, and CD102, non-urea-producing bacteria.

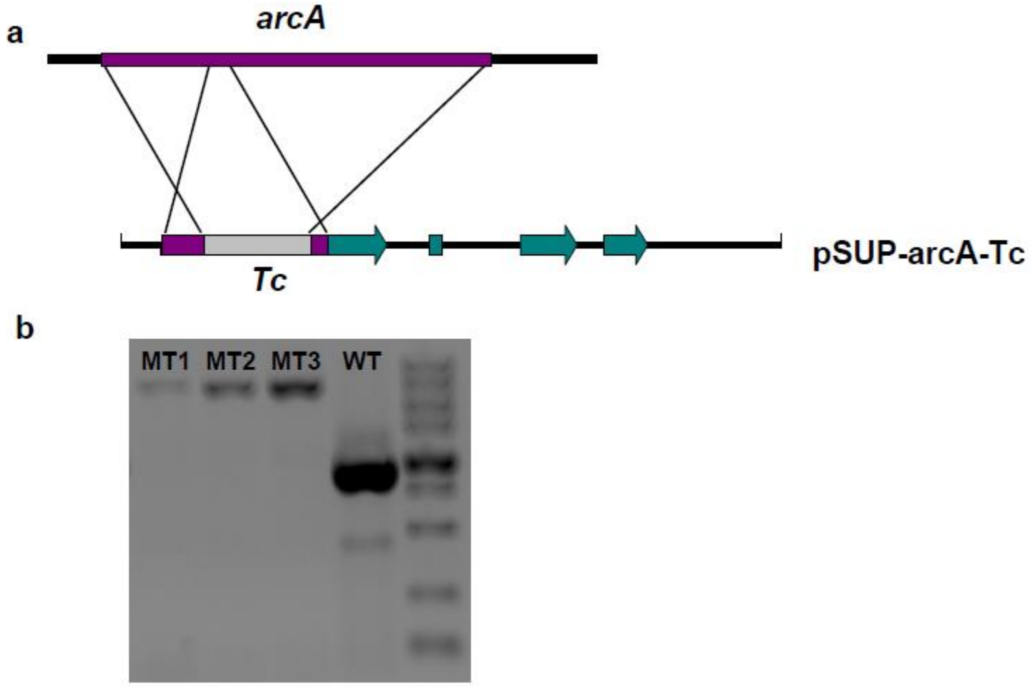

Supplementary Figure 5. PCR analysis of deletion of *arcA* gene in *S. maltophilia*.

(a) Gene deletion strategy. (b) The amplified fragments of *arcA* genes were obtained by PCR from the genomic DNA of wild type (WT), and mutants (MT).

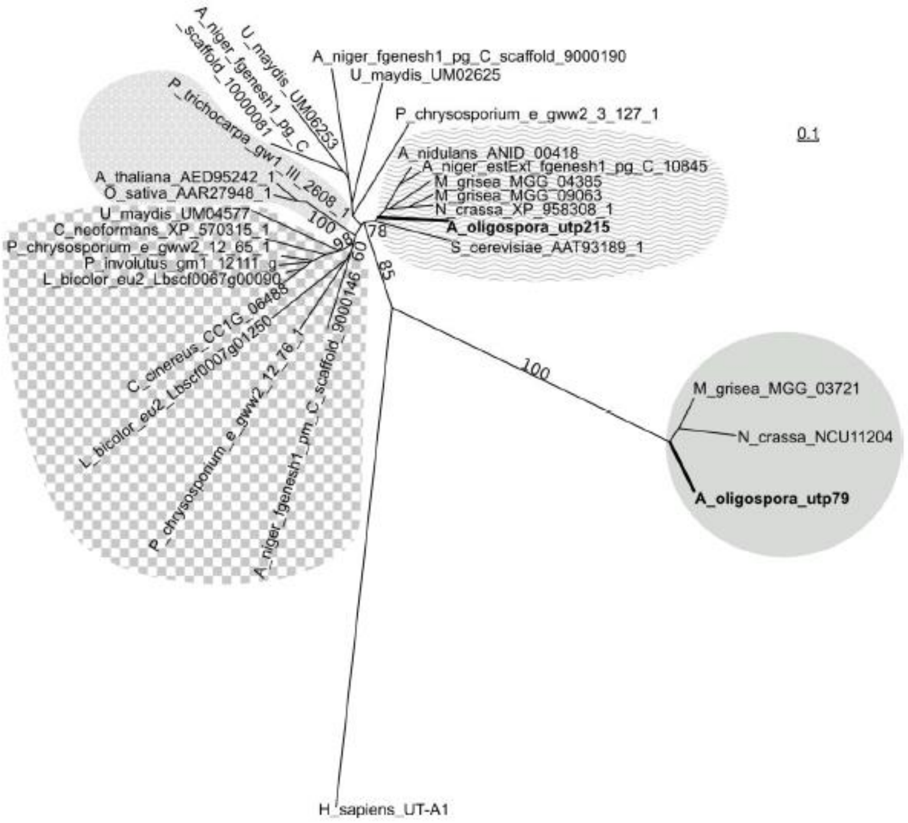

Supplementary Figure 6. The phylogenetic tree of urea transporters in fungi. In the genome of *A. oligospora* ATCC24927, two putative urea transporter genes *utp79* and *utp215* (AOL_S00079g183r and AOL_s00215g323r) were retrieved. Phylogenetic analyses showed that the two urea transporters in *A. oligospora* were clustered into two separate groups.

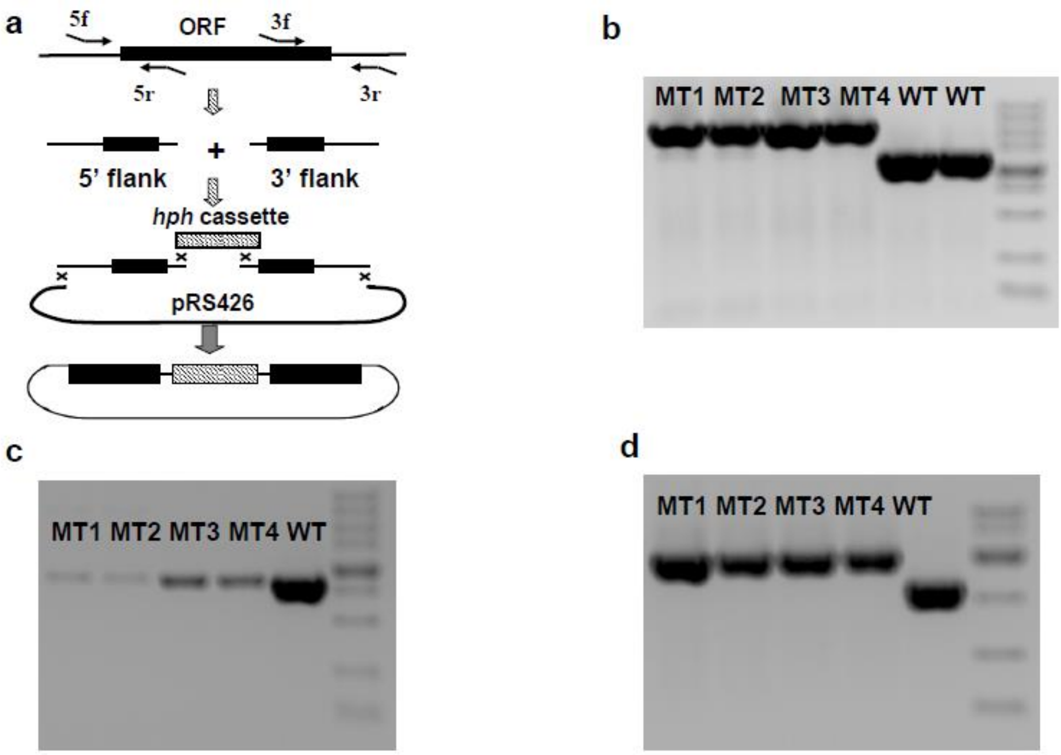

Supplementary Figure 7. PCR analysis of deletion of genes in *A. oligospora.* (**a**) Deletion strategy of genes. (**b-d**) The amplified fragments of genes were obtained by PCR from the genomic DNA of wild type (WT), and mutants (MT). *utp79* (**b**), *utp215* (**c**), *ure1* (**d**).

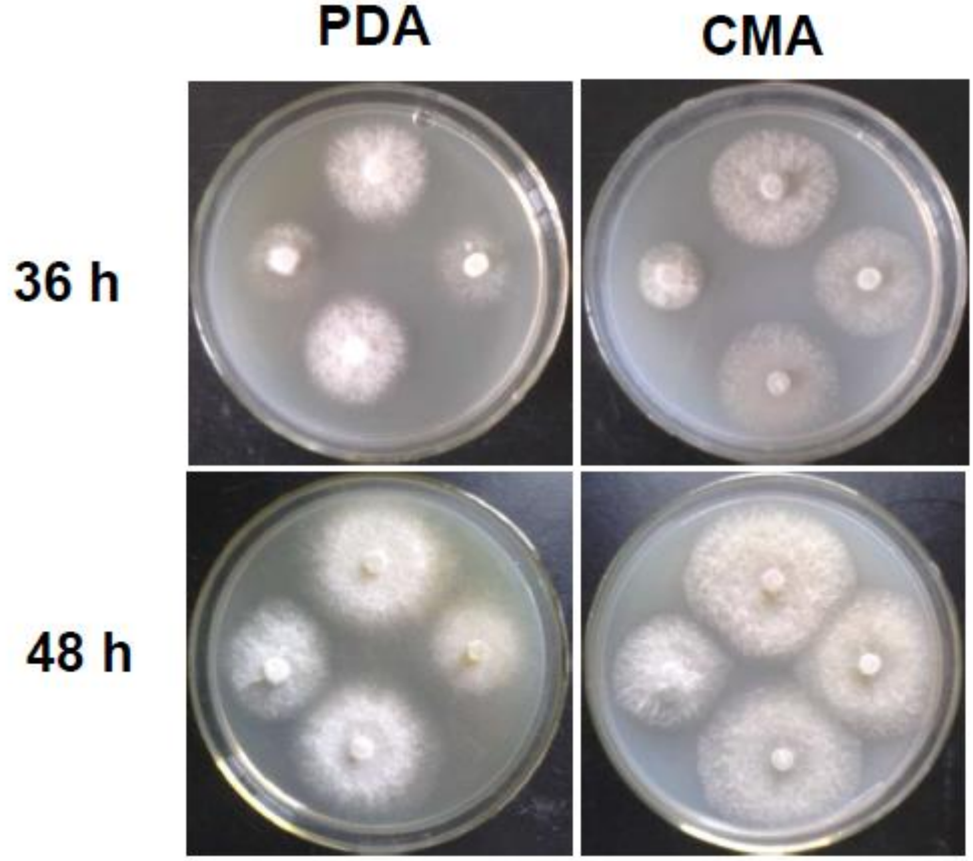

Supplementary Figure 8. The growth phenotype of *A. oligospora* mutants.

Wild-type strain (upper colony), Ao*Δure1* mutant strain (left), Ao*Δutp79* mutant strain (right), and Ao*Δutp215* (lower) were grown on PDA or CMA.

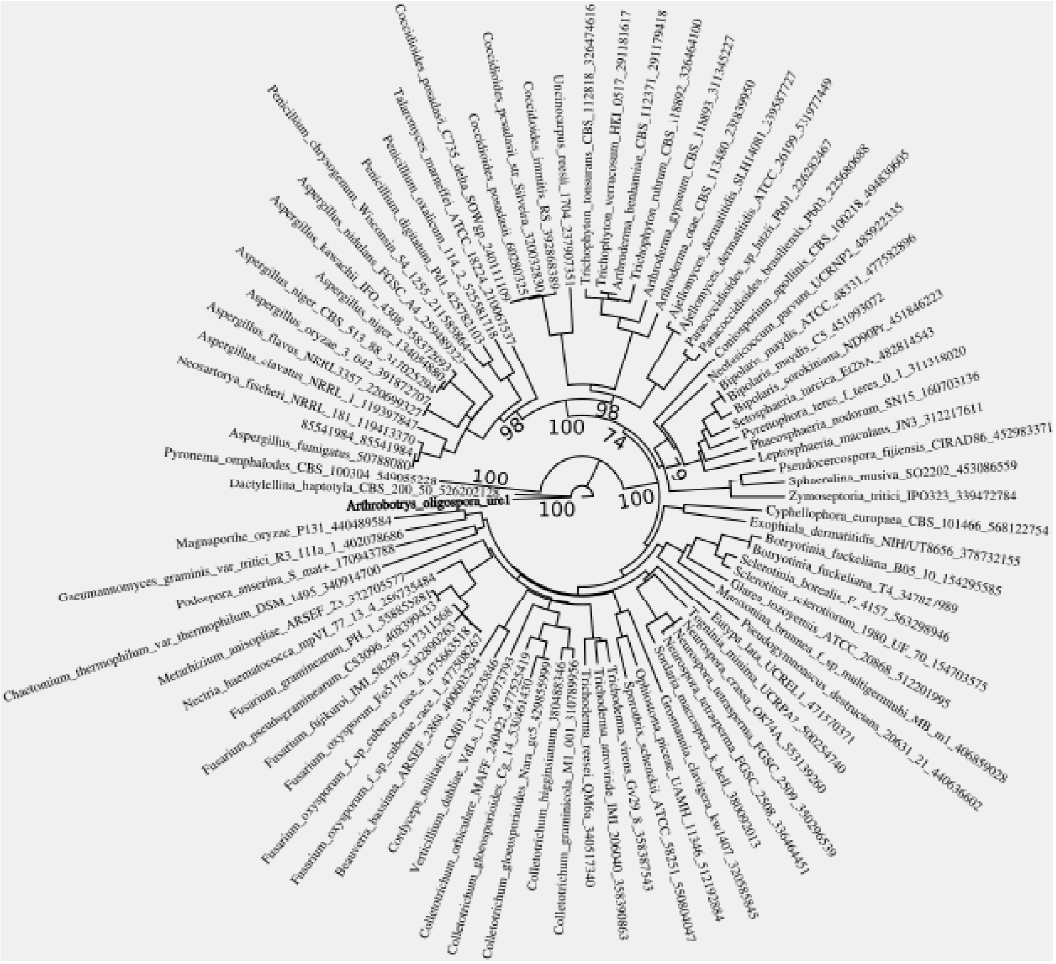

Supplementary Figure 9. The phylogenetic tree of the ureases in fungi. From the genome of *A. oligospora*, a putative urease gene, namely *ure1* (AOL_s00080g26r), was retrieved and analyzed using phylogenetic analyses.

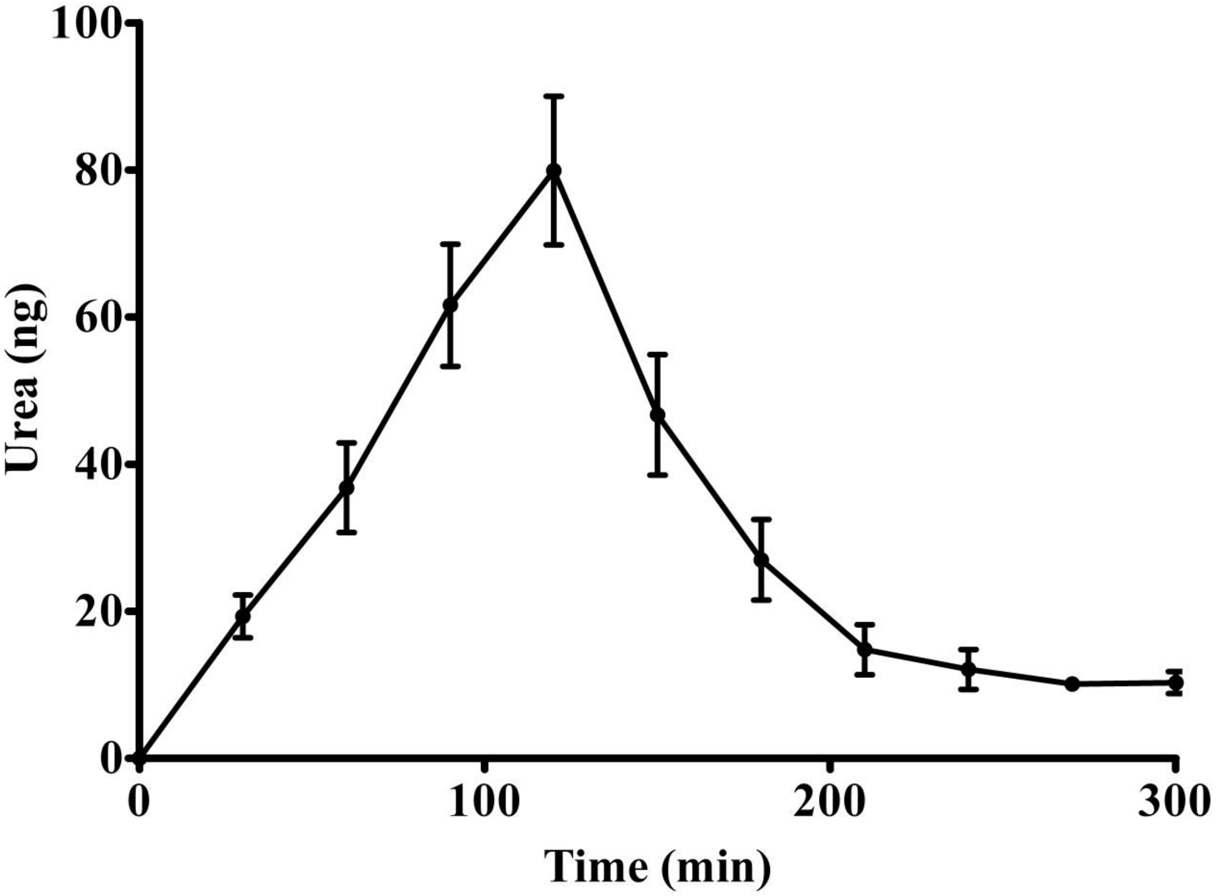

Supplementary Figure 10. Urea diffuses readily through sand. Urea was detected by LC-MS in moist sand at 8 cm from a release point, every 30 min after release.

Supplementary Tables

Supplementary Table 1. The induction of trap formation in *A. oligospora* by the supernatants from bacterial colonies isolated from dung

Number	Genus or species	Induction of trap formation[a]	Production of urea (μg/ml)
CD1	*Bacillus amyloliquefaciens*	0	0
CD2	*Providencia rettgeri*	0	0
CD3	*Acinetobacter* sp.	0	0
CD4	*Acinetobacter* sp.	0	0
CD5	*Acinetobacter* sp.	0	0
CD6	*Aeromonas hydrophila*	104	335.6
CD7	*Brevundimonas bullata*	88	251.2
CD8	*Stenotrophomonas maltophilia*	144	392.7
CD9	*Acinetobacter* sp.	0	0
CD10	*Acinetobacter lwoffii*	99	311.5
CD11	*Acinetobacter* sp.	0	0
CD12	*Alcaligenes faecalis*	103	346.8
CD13	*Bacillus amyloliquefaciens*	0	0
CD14	*Staphylococcus saprophyticus*	0	0
CD15	*Bacillus pumilus*	72	184.6
CD16	*Ochrobactrum intermedium*	69	169.9
CD17	*Acinetobacter johnsonii*	0	0
CD18	*Bacillus* sp.	73	171.2
CD19	*Acinetobacter* sp.	0	0
CD20	*Acinetobacter* sp.	0	0
CD21	*Bacillus cereus*	64	154.2
CD22	*Acinetobacter* sp.	0	0
CD23	*Acinetobacter* sp.	0	0
CD24	*Pseudochrobactrum saccharolyticum*	111	338.9
CD25	*Bacillus marisflavi*	0	0
CD26	*Proteus penneri*	88	261.7
CD27	*Providencia rettgeri*	0	0
CD28	*Acinetobacter* sp.	76	239.7
CD29	*Acinetobacter* sp.	0	0
CD30	*Acinetobacter* sp.	0	0
CD31	*Pseudomonas aeruginosa*	122	301.3
CD32	*Alcaligenes faecalis*	108	307.9
CD33	*Bacillus amyloliquefaciens*	0	0
CD34	*Bacillus pumilus*	51	143.1

CD35	*Bacillus* sp.	0	0
CD36	*Bacillus methylotrophicus*	24	101.2
CD37	*Brevundimonas olei*	97	276.4
CD38	*Acinetobacter* sp.	0	0
CD39	*Acinetobacter junii*	78	241.3
CD40	*Providencia rettgeri*	0	0
CD41	*Stenotrophomonas* sp.	121	389.7
CD42	*Acinetobacter* sp.	86	261.8
CD43	*Acinetobacter* sp.	77	257.9
CD44	*Acinetobacter lwoffii*	0	0
CD45	*Comamonas* sp.	119	346.8
CD46	*Acinetobacter* sp.	63	189.8
CD47	*Bacillus pumilus*	43	100.7
CD48	*Bacillus* sp.	0	0
CD49	*Acinetobacter lwoffii*	69	191.4
CD50	*Acinetobacter* sp.	0	0
CD51	*Bacillus amyloliquefaciens*	0	0
CD52	*Stenotrophomonas maltophilia*	147	421.8
CD53	*Staphylococcus sciuri*	0	0
CD54	*Acinetobacter* sp.	0	0
CD55	*Cronobacter sakazakii*	0	0
CD56	*Acinetobacter* sp.	0	0
CD57	*Bacillus amyloliquefaciens*	0	0
CD58	*Bacillus cereus*	33	79.1
CD59	*Acinetobacter* sp.	0	0
CD60	*Bacillus* sp.	65	191.6
CD61	*Bacillus* sp.	0	0
CD62	*Acinetobacter junii*	0	0
CD63	*Bacillus* sp.	0	0
CD64	*Acinetobacter* sp.	0	0
CD65	*Bacillus altitudinis*	0	0
CD66	*Acinetobacter* sp.	0	0
CD67	*Pseudomonas* sp.	118	311.5
CD68	*Acinetobacter* sp.	0	0
CD69	*Acinetobacter* sp.	0	0
CD70	*Staphylococcus saprophyticus*	0	0
CD71	*Acinetobacter* sp.	0	0
CD72	*Alcaligenes faecalis*	99	289.6
CD73	*Bacillus cereus*	21	70.6
CD74	*Acinetobacter lwoffii*	0	0
CD75	*Bacillus subtilis*	99	205.3
CD76	*Bacillus* sp.	0	0
CD77	*Acinetobacter* sp.	42	132.8

CD78	*Proteus vulgaris*	88	213.1
CD79	*Acinetobacter* sp.	0	0
CD80	*Acinetobacter* sp.	0	0
CD81	*Bacillus methylotrophicus*	59	120.3
CD82	*Escherichia coli*	94	253.2
CD83	*Acinetobacter* sp.	0	0
CD84	*Acinetobacter* sp.	0	0
CD85	*Cronobacter sakazakii*	0	0
CD86	*Bacillus* sp.	0	0
CD87	*Pseudomonas aeruginosa*	109	311.1
CD88	*Acinetobacter* sp.	71	187.6
CD89	*Acinetobacter* sp.	54	163.3
CD90	*Enterococcus casseliflavus*	0	0
CD91	*Escherichia coli*	84	231.8
CD92	*Acinetobacter* sp.	0	0
CD93	*Providencia rettgeri*	0	0
CD94	*Acinetobacter* sp.	49	122.7
CD95	*Bacillus* sp.	65	167.3
CD96	*Wautersiella falsenii*	0	0
CD97	*Staphylococcus sciuri*	0	0
CD98	*Bacillus* sp.	0	0
CD99	*Bacillus* sp.	0	0
CD100	*Acinetobacter junii*	81	204.9
CD101	*Bacillus subtilis*	84	243.8
CD102	*Bacillus safensis*	0	0
CD103	*Stenotrophomonas maltophilia*	139	394.6
CD104	*Acinetobacter towneri*	0	0
CD105	*Bacillus* sp.	0	0
CD106	*Bacillus* sp.	0	0
CD107	*Pseudomonas aeruginosa*	119	328.6
CD108	*Comamonas* sp.	102	330.4
CD109	*Acinetobacter junii*	0	0
CD110	*Klebsiella* sp.	113	315.8
CD111	*Bacillus* sp.	0	0
CD112	*Proteus penneri*	99	189.7
CD113	*Enterococcus mundtii*	0	0
CD114	*Acinetobacter* sp.	64	209.7
CD115	*Acinetobacter* sp.	0	0
CD116	*Acinetobacter* sp.	67	204.6
CD117	*Bacillus cereus*	51	161.6
CD118	*Bacillus pumilus*	0	0
CD119	*Acinetobacter* sp.	0	0
CD120	*Comamonas* sp.	126	330.9

CD121	*Acinetobacter* sp.	0	0
CD122	*Proteus* sp.	0	0
CD123	*Acinetobacter* sp.	43	144.9
CD124	*Bacillus* sp.	64	196.5
CD125	*Bacillus cereus*	56	156.8
CD126	*Pseudomonas stutzeri*	95	289.4

[a] After bacteria were grown in LB medium on a rotary shaker at 30°C for four days, fermentation supernatant was collected and placed on a water agar plate. Conidial suspension with a concentration of about 5×10^4 conidia/ml was spread over the plate and incubated at 25°C. Trap numbers were counted in 10 low-power light microscope fields, and reported as the average number per field of observation.

Supplementary Table 2. The induction of trap formation by urea in different nematode-trapping fungi.

Number[a]	Fungal species	Types of trap structures	Trap formation
YMF1.00553	*Drechslerella doedycoides*	constricting rings	×
YMF1.01480	*Drechslerella coelobrocha*	constricting rings	×
YMF1.00018	*Arthrobotrys pyriformis*	adhesive nets	×
YMF1.00033	*Dactylellina gephyrophaga*	adhesive columns	×
YMF1.03529	*Arthrobotrys polycephala*	adhesive nets	×
YMF1.01420	*Arthrobotrys oudemansii*	adhesive nets	×
YMF1.01402	*Arthrobotrys longiphora*	adhesive nets	×
YMF1.00010	*Arthrobotrys dendroides*	adhesive nets	×
YMF1.00537	*Arthrobotrys globosporum*	adhesive nets	×
YMF1.01829	*Drechslerella brochopaga*	constricting rings	√
YMF1.03412	*Arthrobotrys cystosporia*	adhesive nets	×
YMF1.00014	*Arthrobotrys guizhouensis*	adhesive nets	√
YMF1.00550	*Drechslerella heterospora*	constricting rings	×
YMF1.00028	*Arthrobotrys microscaphoides*	adhesive nets	√
YMF1.03291	*Arthrobotrys globosporum*	adhesive nets	×
YMF1.01883	*Arthrobotrys oligospora*	adhesive nets	√
YMF1.00040	*Arthrobotrys rutgeriense*	adhesive nets	×
YMF1.00583	*Drechslerella effusa*	constricting rings	×
YMF1.01424	*Arthrobotrys cladodes*	adhesive nets	×
YMF1.01460	*Dactylellina leptospora*	adhesive knobs	×
YMF1.01879	*Arthrobotrys conoides*	adhesive nets	√
YMF1.01429	*Drechslerella bembicodes*	constricting rings	×
YMF1.01474	*Dactylellina phymatopaga*	adhesive knobs	√
YMF1.01427	*Drechslerella aphrobrocha*	constricting rings	×
YMF1.01472	*Dactylellina cionopaga*	adhesive columns	√
YMF1.00025	*Arthrobotrys sinensis*	adhesive nets	×
YMF1.00571	*Arthrobotrys dianchiensis*	adhesive nets	×
YMF1.03316	*Arthrobotrys oviformis*	adhesive nets	×
YMF1.00593	*Arthrobotrys yunnanensis*	adhesive nets	×
YMF1.01477	*Arthrobotrys multiformis*	adhesive nets	×
YMF1.03468	*Dactylellina lysipaga*	adhesive knobs	×

[a] The accession numbers from the Southwest Natural Germplasm Center for Microbial Resources. √, Trap formation is observed; ×, Trap formation is not detected.
